# Aortic Root Replacement for Destructive Endocarditis - Clinic and Microbiology

**DOI:** 10.21470/1678-9741-2020-0412

**Published:** 2021

**Authors:** Marcin P. Szczechowicz, Alexander Weymann, Sabreen Mkalaluh, Ahmed Mashhour, Konstantin Zhigalov, Michel Pompeu B. O. Sá, Alina Zubarevich, Jerry Easo

**Affiliations:** 1 Department of Cardiac Surgery, University of Duisburg-Essen, Essen, Germany.; 2 Department of Cardiac Surgery, Oldenburg University Hospital, Oldenburg, Germany.; 3 Division of Cardiovascular Surgery of Pronto-Socorro Cardiológico Universitário de Pernambuco - PROCAPE, Universidade de Pernambuco, Recife, Pernambuco, Brazil.

**Keywords:** Endocarditis, Bacterial, Coagulase, Incidence, Anti-Bacterial Agents, Prognosis, Reinfection, Delayed Diagnosis, Risk Factors

## Abstract

**Introduction:**

Destructive aortic root endocarditis is associated with high mortality rates. The objective of this article was to characterize the clinical and microbiological profiles of these patients, especially concerning an already implanted aortic valve prosthesis. We also focused on prognostic factors.

**Methods:**

Eighty patients underwent aortic root replacement due to destructive endocarditis from 1999 to 2018 in our institution. We analyzed their pre, intra, and postoperative data, outcomes, and predictors of mortality.

**Results:**

Thirty-one patients had native valve endocarditis (NVE), eight patients had early-onset prosthetic valve endocarditis (PVE), and 41 patients had late-onset PVE. *Streptococcus* was found in 19.4% of NVE cases and no PVE case. Coagulase-negative *Staphylococcus* was responsible for 62.5% of the cases of early-onset PVE. Thirty-four (42.5%) patients had received inappropriate antibiotics before admission. No microorganism was associated with higher risk of mortality. Aortoventricular dehiscence was identified as an independent risk factor of mortality along with PVE, concomitant bypass surgery, and delayed diagnosis. The incidence of postoperative complications was similar in all three groups. Rates of long-term survival (*P*=0.044) and freedom from the composite endpoint (*P*=0.024) defined as death, stroke, aortic valve reinfection, and aortic valve reoperation were the lowest within the NVE group and the highest among the PVE patients.

**Conclusion:**

In endocarditis, prolonged diagnostics, inadequate antimicrobial treatment, and late surgery led to destructive local complications and worsened the prognosis. PVE is associated with higher mortality than NVE.

**Table:** 

Abbreviations, acronyms & symbols	
BCNIE	= Blood culture-negative IE
CABG	= Coronary artery bypass grafting
CI	= Confidence intervals
CoNS	= Coagulase-negative Staphylococci
CPB	= Cardiopulmonary bypass
ECMO	= Extracorporeal membrane oxygenation
FFCE	= Freedom from the composite endpoint
HACEK	= Hemophilus spp., Aggregatibacter actinomycetemcomitans, Cardiobacterium hominis, Eikenella corrodens, and Kingella kingae
HR	= Hazard ratios
IE	= Infective endocarditis
NVE	= Native valve endocarditis
OR	= Odds ratios
PVE	= Prosthetic valve endocarditis

## INTRODUCTION

Despite new antimicrobial agents and advancements in surgical therapy, the mortality rate of infective endocarditis (IE) remains very high ^[1]^. Worldwide, the incidence of IE varies from 1.5 to 11.6 cases/100,000 person-years ^[2]^. Over 90% of all cases are infections of the left-sided heart valves ^[3]^. The IE of prosthetic aortic valves has a much higher incidence at the level of 70/10,000 person-years with a described five-year mortality of 18.3% and 10-year mortality of 43.6%. The history of prosthetic heart valve implantation increases the cumulative incidence of IE up to 5% at 10 years and is one of the major risk factors of IE ^[1]^. The likelihood of bioprosthetic valve infection has been described as twice as high when compared to mechanical prosthetic valves ^[4]^.

Prosthetic valve endocarditis (PVE) diagnosis is usually delayed because it is often more challenging than the diagnosis of native valve endocarditis (NVE), thus giving the infectious process more chance for annular disruption leading to periannular disease. If the valve is affected in the aortic position, the locally uncontrolled infection can destroy surrounding structures leading to life-threatening complications, such as the formation of abscesses, aortic pseudoaneurysms, intracardiac shunts, or aortoventricular dehiscence ^[5]^. Generally, radical surgical debridement, reconstruction of the annulus, and valve replacement can be attempted, but the standardized replacement of the whole aortic root is preferred in most cases ^[6,7]^.

We analyzed consecutive cases of aortic root endocarditis, which had to be treated with full root replacement. The study aimed to characterize these patients and to compare their microbiological profiles, postoperative courses, and survival rates. Also, we identified potential risk factors of short- and long-term mortality.

## METHODS

### Patients

A total of 483 patients underwent aortic valve or root surgery due to IE in our clinic between 1999 and 2018. Among them, 80 patients underwent total aortic root replacement due to local complications of IE (such as intracardiac shunt, large abscess, inflammatory aortic aneurysm, chronic dissection, or severe aortoventricular dehiscence), poor quality of infected aortic root tissue, or extensive infection of already implanted root prosthesis. These 80 subjects were included in our study. No patients were excluded.

We divided our sample into three subgroups, which were considered as independent samples: patients with NVE, patients with early-onset PVE, and patients with late-onset PVE; and we analyzed pre, intra, postoperative, and follow-up data.

### Definitions

PVE occurring within 12 months after valve implantation is defined as early-onset PVE. If the infection occurs later than one year after surgery, it is defined as late-onset PVE ^[8]^. These definitions are used in this report. Some authors have defined only the reinfections occurring within the first 120 or even 60 days after surgery as early-onset PVE and those occurring from this time to the 365^th^ day as intermediate-onset PVE. The latter definition mixes clinical and microbiological profiles of both early- and late-onset PVEs ^[8,9]^.

### Statistical Analysis

Categorical variables were presented as absolute values with percentages, and their distributions were compared between the groups using the Chi-square test. Continuous variables were presented as median values with quartiles. Because of the relatively low number of cases, we assumed a lack of normal distributions. In order to compare the means between the groups, we performed the Kruskal-Wallis test. If the results were statistically significant, a post-hoc Dunn’s test was done to examine the differences between data distributions.

Survival and freedom from the composite endpoint (FFCE), which was defined as death, stroke, aortic valve reinfection, and aortic valve reoperation for any cause, were analyzed with the Kaplan-Meier method and compared between the groups generally and pairwise with the use of the log-rank test. We used a univariate logistic regression to identify the risk factors of 30-day mortality within the whole sample. The results are presented as odds ratios (ORs) with 95% confidence intervals (CIs). Univariate proportional hazard regression was used to identify the risk factors of long-term mortality within the whole sample. The results are shown as hazard ratios (HRs) with 95% CIs. The incidence rates of aortic valve reoperation and FFCE within the follow-up periods were compared with the use of the polynomial multiplication method and are presented as a number of events/100 patients-years. Overall, *P*-values < 0.05 were considered as statistically significant. For the statistical analysis, we used the R software v.3.4.3 (R Foundation for Statistical Computing, Vienna, Austria) in addition to the IBM Corp. Released 2017, IBM SPSS Statistics for Windows, Version 25, Armonk, NY: IBM Corp.

## RESULTS

### Preoperative Characteristics

Among our 80 root endocarditis patients, 31 (38.75%) had NVE, eight (10%) had early-onset PVE, and 41 (51.25%) had late-onset PVE. The median age at the time of surgery was 64 years. Patients with NVE were significantly younger than those with early (*P*=0.044) and late (*P*=0.036) PVE. Eleven patients (13.8%) were female. Among the PVE patients, the median time from the prior aortic valve implantation to surgery for endocarditis was 4.2 (1.6 to 7.6) years. The demographics and preoperative characteristics of our sample are presented in Table 1.

**Table 1:** Preoperative characteristics and comorbidities.

Characteristics	Native valve endocarditis	Early-onset prosthetic valve endocarditis	Late-onset prosthetic valve endocarditis	*P*-value
N	31 (38.75%)	8 (10%)	41 (51.25%)	
Female	3 (9.7%)	0	8 (19.5%)	0.269
Age (years)	55 (47 to 69)	71.5 (62 to 74.8)	66 (55 to 72)	0.010
Previous heart surgery	3 (9.7%)	8 (100%)	41 (100%)	-
Aortic valve surgery	0	8 (100%)	41 (100%)	< 0.001
Mechanical aortic valve prosthesis	0	2 (25%)	20 (48.8%)	< 0.001
Biological aortic valve prosthesis	0	6 (75%)	21 (51.2%)	< 0.001
Coronary artery bypass grafting	0	0	10 (24.4%)	0.004
Time from prior aortic valve surgery to redo surgery for destructive endocarditis	-	115 days (101 to 222)	5.1 years (3.2 to 8.9)	< 0.001
Relevant coronary artery disease	4 (12.9%)	1 (12.5%)	11 (26.8%)	0.293

### Surgical Data

In all cases, a standard approach with median sternotomy, cardiopulmonary bypass (CPB), and cardioplegic cardiac arrest was used. Median surgery time and median CPB time were understandably significantly shorter within the NVE group than within the late-onset PVE group (*P*=0.036). The aortoventricular dehiscence was intraoperatively diagnosed only in one patient (3.2%) with NVE, but in five (62.5%) cases of early-PVE and in 14 (34.1%) cases of late-PVE (*P*<0.001). The frequency of other endocarditis-related intraoperative findings, such as fistula or abscess, did not differ significantly between the groups (*P*=0.939 and *P*=0.274, respectively). Fourteen (17.5%) patients underwent mitral valve replacement due to IE (neither commando nor hemi-commando procedures) and in 17 (21.25%) cases, coronary artery bypass grafting (CABG) was performed. In nine (11.25%) cases, bypass surgery had to be performed as a bailout procedure, etiologically due to severely destructed coronary ostia. In two (25%) patients with early-onset PVE, extracorporeal membrane oxygenation (ECMO) was implanted as short-term circulatory support. ECMO was also necessary in two (6.5%) cases of NVE but in no cases of late-PVE. Detailed surgical data are presented in Table 2.

**Table 2:** Surgical data.

Characteristics	Native valve endocarditis	Early-onset prosthetic valve endocarditis	Late-onset prosthetic valve endocarditis	*P*-value
N	31 (38.75%)	8 (10%)	41 (51.25%)	
Surgery time (min)	209 (160 to 302)	263 (218 to 438)	303 (251 to 390)	0.004
Cardiopulmonary bypass time (min)	127 (92 to 231)	161 (135 to 293)	195 (147 to 249)	0.040
Cross-clamping time (min)	100 (71 to 135)	118 (99 to 144)	128 (102 to 148)	0.065
Periannular complications				
Fistula	3 (9.7%)	1 (12.5%)	5 (12.2%)	0.939
Abscess	13 (41.9%)	2 (25%)	22 (53.7%)	0.274
Aortoventricular dehiscence	1 (3.2%)	5 (62.5%)	14 (34.1%)	< 0.001
Type of implanted aortic root prosthesis				
Porcine aortic root xenograft	22 (71%)	6 (75%)	25 (61%)	0.727
Biological valve conduit	5 (16.1%)	0	8 (19.5%)	
Mechanical valve conduit	2 (6.5%)	1 (12.5%)	2 (4.9%)	
Homograft valve	2 (6.5%)	1 (12.5%)	6 (14.6%)	
Concomitant procedures	14 (45.2%)	3 (37.5%)	25 (61%)	0.276
Mitral valve replacement	7 (22.6%)	1 (12.5%)	6 (14.6%)	0.629
Replacement of the ascending aorta	2 (6.5%)	2 (25%)	11 (26.8%)	0.080
Coronary artery bypass grafting	5 (16.1%)	0	12 (29.3%)	0.121
Among them: bailout bypass	3 (9.7%)	0	6 (14.6%)	0.458

###  

### Microbiology

*Streptococcus* spp. were identified as the cause for infection in six (19.4%) cases of NVE and no case of PVE (*P*=0.006). Coagulase-negative *Staphylococci* (CoNS) were responsible for five (62.5%) of the early-onset PVE cases and their contributions to NVE (n=8, 19.5%) and late-onset PVE (n=8, 25.8%) were significantly lower (*P*=0.041). *Enterococci* and *Staphylococcus aureus* were found mostly in late-onset PVE patients; however, no statistically significant predominance (*P*=0.073 and *P*=0.093, respectively) was noted. All in all, the *Staphylococcus* spp. was the etiological agent in 29 (36.3%) cases with equal distribution beyond the groups. Other microorganisms were grown with similar frequency in all three groups. In 27 (33.75%) cases, preoperative blood cultures and postoperative valve cultures were negative. Culture-negative cases were equally distributed among the groups. The prevalence of infections caused by resistant bacteria was equally distributed (*P*=0.693). Fourteen (17.5%) patients were admitted without any antibiotic treatment, and 20 (25%) had only empirical monotherapy at the time of admission, which resulted in 34 (42.5%) patients admitted without proper diagnostics and adequate antimicrobial therapy. The microbiological characteristics of our sample are listed in Table 3.

**Table 3:** Microbiological characteristics.

Characteristics	Native valve endocarditis	Early-onset prosthetic valve endocarditis	Late-onset prosthetic valve endocarditis	*P*-value
N	31 (38.75%)	8 (10%)	41 (51.25%)	
*Staphylococcus* spp.	9 (29%)	5 (62.5%)	15 (36.6%)	0.214
Methicillin-resistant *Staphylococcus* spp.	3 (9.7%)	2 (25%)	5 (12.2%)	0.504
*S. aureus*	1 (3.2%)	0	7 (17.1%)	0.093
Coagulase-negative *Staphylococcus*	8 (25.8%)	5 (62.5%)	8 (19.5%)	0.041
*Streptococcus* Type A or B	6 (19.4%)	0	0	0.006
*Enterococcus*	1 (3.2%)	1 (12.5%)	9 (22%)	0.073
Another Gram-positive	3 (9.7%)	0	2 (4.9%)	0.526
Gram-negative	1 (3.2%)	0	0	0.449
*Candida*	0	0	1 (2.4%)	0.618
Unknown	11 (35.5%)	2 (25%)	14 (34.1%)	0.853
Resistant microorganisms	8 (25.8%)	3 (37.5%)	14 (34.15)	0.693
No antibiotics at admission	5 (16.1%)	3 (37.5%)	6 (14.6%)	0.288
Antimicrobial monotherapy at admission	10 (32.3%)	1 (12.5%)	9 (22%)	0.419

### Postoperative Courses

Detailed postoperative data and incidences of postoperative adverse events are presented in Table 4.

**Table 4:** Postoperative characteristics.

Characteristics	Native valve endocarditis	Early-onset prosthetic valve endocarditis	Late-onset prosthetic valve endocarditis	*P*-value
N	31 (38.75%)	8 (10%)	41 (51.25%)	
Acute kidney injury	3 (9.7%)	2 (25%)	11 (26.8%)	0.184
Resternotomy due to bleeding	6 (19.4%)	3 (37.5%)	4 (9.8%)	0.126
Stroke	0	0	2 (4.9%)	0.377
Respiratory failure	5 (16.1%)	4 (50%)	13 (31.7%)	0.110
Low output syndrome	2 (6.5%)	2 (25%)	4 (9.8%)	0.296
Pacemaker implantation	2 (6.5%)	1 (12.5%)	5 (12.2%)	0.702
Packed red cells transfusion (ml)	900 (600 to 2250)	3900 (450 to 6975)	1350 (600 to 3000)	0.497
Mechanical ventilation (hours)	5 (2 to 39)	147 (9 to 463)	24 (3 to 84)	0.045
Intensive care unit length of stay (days)	3 (1 to 6)	7 (3 to 14)	3 (2 to 9)	0.569
In-hospital length of stay (days)	8 (6 to 17)	10 (6 to 14)	9 (7 to 14)	0.826
30-day mortality	3 (9.7%)	3 (37.5%)	16 (39%)	0.018

### Follow-up

**Fig. 1:**
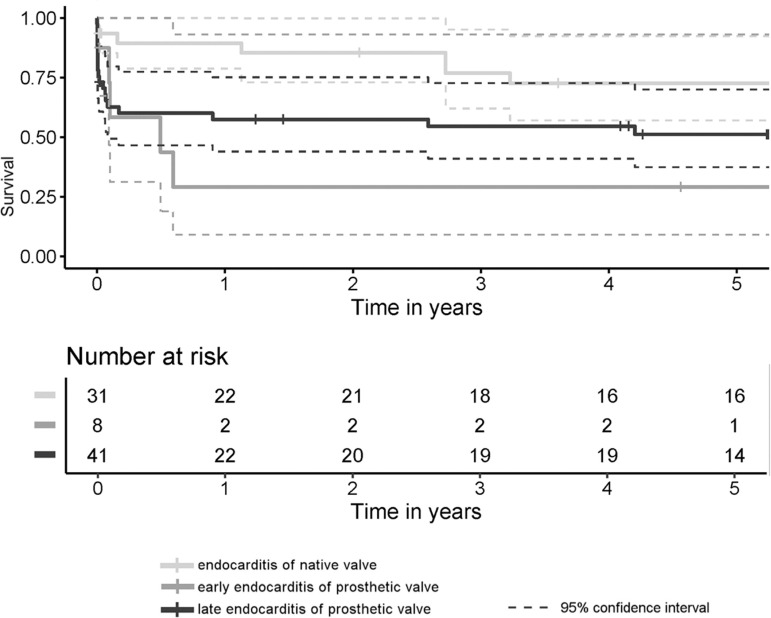
Cumulative survival of all three analyzed groups. The survival profiles were compared with log-rank test, generally (P=0.044) and pairwise: native valve endocarditis (NVE) vs. early-onset prosthetic valve endocarditis (PVE) (P=0.008), NVE vs. late-onset PVE (P=0.039), and early- vs. late-onset PVE (P=0.499).

**Fig. 2:**
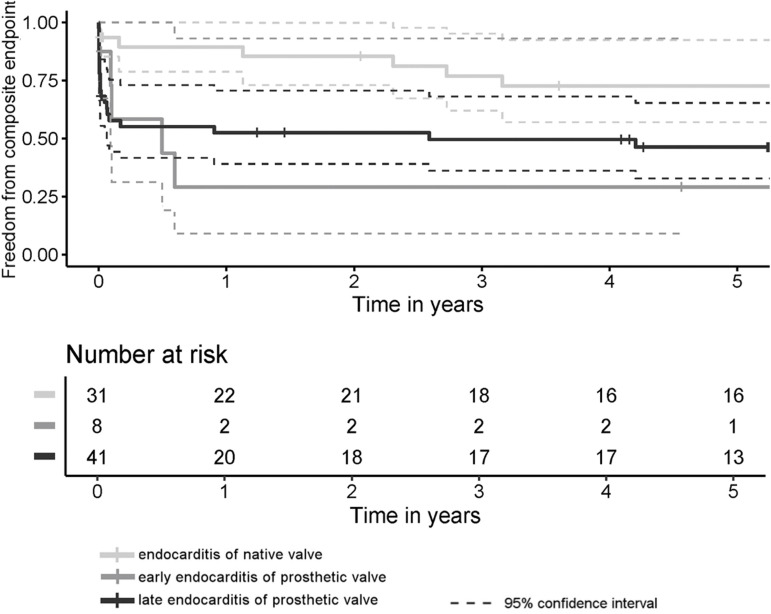
Cumulative freedom from composite endpoint (stroke, aortic valve reinfection, and aortic valve reoperation for any cause) within all three analyzed groups. Comparison with log-rank test, generally (P=0.024) and pairwise: native valve endocarditis (NVE) vs. early-onset prosthetic valve endocarditis (PVE) (P=0.008), NVE vs. late-onset PVE (P=0.013), and late- vs. early-onset PVE (P=0.133).

Thirty-day mortality rate was 9.7% among the NVE patients (n=3), 37.5% (n=3) among the early-onset PVE patients, and 39% (n=16) within the late-onset PVE group. The differences between NVE and early-onset PVE and between NVE and late-onset PVE were statistically significant (*P*=0.026 and *P*=0.022, respectively). The early- and late-onset PVE cases did not differ significantly from each other (*P*=0.798). The median follow-up time was 4.9 (0.8-8.9) years. The five-year survival rates were better after NVE than after late-onset PVE but worst after the early-onset PVE (Figure 1). The incidence of FFCE within five years after surgery was distributed in a similar manner as seen with the survival functions, but the differences were slightly more significant (Figure 2). There were four cases of reoperations for valve reinfections during follow-up (7.4% of discharged patients), and all of them occurred within the NVE group. Two of them died within the postoperative course after the repeat surgery. The median time from the current surgery to the repeat surgery was 6.9 years, and there were no cases of early-onset PVE among these patients.

### Mortality Predictors

We were able to identify strong risk factors, such as aortoventricular dehiscence and bypass surgery, especially bailout CABG, for short- and long-term mortalities. Concomitant mitral valve surgery was not associated with increased risks. PVE was an independent risk factor of 30-day mortality. Patients who had not received any antimicrobial therapy at admission were also at higher risk of death within 30 postoperative days. No specific etiological agent was associated with increased or decreased mortality. All examined variables with ORs and HRs with 95% CIs are presented in Table 5.

**Table 5:** Independent risk factors of mortality

Characteristics	30-day mortality - univariate logistic regression		Long-term mortality - univariate proportional hazard regression	
	Odds ratio with 95% confidence interval	*P*-value	Hazard ratio with 95% confidence interval	*P*-value
Age (years)	1.03 (0.00 to 1.07)	0.143	1.02 (0.99 to 1.05)	0.153
History of cardiac surgery	4.8 (1.3 to 18.0)	0.020	1.5 (0.7 to 3)	0.289
History of aortic valve surgery	5.1 (1.4 to 19.3)	0.015	1.6 (0.8 to 3.2)	0.206
PVE	5.9 (1.6 to 22.0)	0.008	1.9 (0.9 to 3.9)	0.083
Fistula	4.0 (0.96 to 16.5)	0.058	2.2 (0.9 to 5.4)	0.079
Abscess	0.96 (0.36 to 2.6)	0.930	0.8 (0.4 to 1.6)	0.571
Aortoventricular dehiscence	5.4 (1.8 to 16.3)	0.002	2.4 (1.2 to 4.7)	0.014
Concomitant coronary artery bypass grafting	8.7 (2.6 to 28.4)	<0.001	3.8 (1.8 to 7.8)	<0.001
Bailout bypass surgery	13.1 (2.5 to 69.0)	0.003	4.3 (1.9 to 9.9)	<0.001
Unknown microorganism	0.9 (0.3 to 2.5)	0.822	0.9 (0.5 to 1.9)	0.866
Resistant microorganisms	0.8 (0.3 to 2.3)	0.637	1.1 (0.5 to 2.2)	0.871
No antibiotics at admission	5.0 (1.5 to 16.6)	0.010	2 (0.9 to 4.4)	0.086

PVE=prosthetic valve endocarditis

## DISCUSSION

Late-onset PVE of biological prostheses is most likely to be limited only to the valve leaflets. Early-onset PVE and PVE of mechanical valves often involve the sewing ring and periannular structures thus leading to the formation of abscesses, fistulas, sewing ring detachments, and other forms of excavating root pathology ^[10]^. Echocardiography is much more challenging in PVE patients and often produces a false negative. In cases of PVE, blood cultures are also prone to false interpretation, often being less sensitive. These difficulties delay the necessary diagnosis required for rapid and adequate treatment of this high-risk patient cohort ^[1]^.

Early surgical intervention in IE reduces the mortality rates but only can be performed in patients with adequate diagnosis, which is often delayed, followed by an exacerbation of the course, increases in periannular complications, and worsening of the prognosis ^[11-13]^. In a large multicenter study by Erdem et al. ^[14]^, which included 867 patients who were hospitalized due to IE, 72.8% of patients were identified as having NVE ^[14]^. In our sample, the patients with PVE were the majority (61%). Other authors that analyzed the destructive form of IE described also this shift toward PVE within cohorts with locally uncontrolled infections ^[15,16]^.

Other factors that have been shown to cause an increase in the risk of local complications are the high virulence of microorganisms and the selection of inappropriate antibiotics ^[17]^. Thirty-four (42.5%) of our patients received either inappropriate antimicrobial therapy or no antibiotics at all upon admission, most likely due to late or wrong diagnosis. Patients who were misdiagnosed and received no antibiotics upon admission had a five times higher risk of 30-day mortality. We suggest that delayed diagnosis contributed significantly to the destruction of periannular tissues in these cases.

An important factor that makes the diagnose of IE more difficult is a negative blood culture ^[13]^. Blood culture-negative IE (BCNIE) is either caused by intracellular microorganisms (*Bartonella*, *Coxiella*, *Tropheryma*, or others) or by bacteria that need prolonged incubation or incubation in special settings (*Hemophilus* spp., *Aggregatibacter actinomycetemcomitans*, *Cardiobacterium* *hominis*, *Eikenella corrodens*, and *Kingella kingae* [HACEK], *Propionibacterium*, *Candida*, or others) ^[18]^. It can be also caused by agents that are typical for IE, but the blood cultures were usually taken after empirical antibiotic administration ^[19]^. Some reports have identified inappropriate prescription of antibiotics as a potential cause of delayed diagnosis ^[20]^. The prevalence of BCNIE in our sample was 33.75%, which was much more than in samples that found it not only in patients with destructive endocarditis (Ferrera et al. ^[13]^: 14.2%, Erdem et al. ^[14]^: 21.6%).

The time frames that define early- and late-onset PVE are crucial for the choice of the empirical antimicrobial treatment but are an arbitral concept and useful only if the blood or valve cultures are negative or pending ^[9]^. In a prospective study by Siciliano et al., 77% of PVE occurred within the first 120 days after valve surgery, and the most frequently isolated microorganisms were CoNS (45% of cases) followed by *S. aureus*, *Enterococcus* spp., Gram-negative bacilli, *Streptococcus* spp., fungi, and HACEK. The authors observed a distinct shift toward *Streptococcus* spp. and fewer infections with resistant microorganisms after the 120^th^ postoperative day ^[8]^. Their sample, however, contained only PVE patients. In our material, the surgery for early-onset PVE was in most cases performed up to the eighth month after the initial aortic valve surgery. Among our patients with early-onset PVE, an even higher percentage was diagnosed with CoNS, but there were no cases of streptococcal PVE even after one year postoperatively. The only streptococcal infections in our sample were six (19.4%) cases of NVE. Interestingly, all these *Streptococci* were methicillin-sensitive, but the diagnoses were delayed or incorrect in these cases, and only one of these patients was admitted to our clinic with appropriate antimicrobial therapy. We also observed more infections with sensitive microorganisms in the late-onset than in the early-onset PVE cohort; however, these differences were not statistically significant.

Elgalad et al. described a cohort of 168 patients with destructive aortic valve IE, who underwent surgery with diverse methods (valve replacement, annulus reconstruction, stentless valve, homograft, or composite graft implantation). In their study, CoNS were isolated in 17.9% of patients, while *S. aureus* occurred in 16.6% and was associated with aortic root abscess and need for extensive root reconstruction or replacement. That finding indicated that *Staphylococcus* spp. was responsible for 34.5% of IE cases, which is comparable to our sample. However, only 44.6% of their patients had PVE (without distinction between early- and late-onset types), and all other underwent surgery due to NVE ^[15]^. The periannular extension of infection was in those patients probably less advanced than in our sample because only 49% of them needed aortic root replacement. Their microbiological profiles resemble our NVE and late-onset PVE cohorts.

Pettersson et al. analyzed 146 patients who underwent double (aortic and mitral) valve replacement for IE either with or without cardiac skeleton reconstruction. In this sample, 52% of patients had PVE and 48% had NVE, but *Staphylococcus* spp. was the most common microorganism and was found in 50% cases. *S. aureus* was significantly more frequently isolated from patients who suffered from PVE and needed the commando procedure due to the destruction of the intervalvular fibrous body ^[16]^. However, their sample contained only patients with double-valve endocarditis; therefore, its microbiological profile could have differed from ours.

Castillo et al. ^[21]^ pointed out that late-onset PVE can be treated successfully with similar success rates as NVE, whereas early-onset PVE is typically fulminant and has a poor prognosis despite optimal surgical treatment. In our study, the courses and outcomes differed between groups with PVE and NVE and did not differ between early- and late-onset PVE; however, we analyzed only patients with the most severely destroyed aortic root.

Concomitant CABG is considered to increase the perioperative risk in IE patients ^[22]^. Also, in our case series, the necessity for concomitant bypass surgery was a strong predictor of short- and long-term mortality. Understandably, the bailout CABG is even a stronger predictor of mortality.

### Limitations

Although our sample is one of the biggest cohorts of patients who underwent aortic root replacement due to severe destructive endocarditis ever described, it consists only of 80 patients, being relatively small. The retrospective enrollment of patients from a single center is the most important limitation of our study.

## CONCLUSION

PVE is associated with higher mortality than NVE. Negative blood cultures due to inappropriate empirical antibiotics use can prolong the diagnostics of IE. This delays administration of adequate antimicrobial and surgical therapy and leads to destructive local complications and worse prognosis. A history of incorrect empirical therapy with antibiotics is often seen in patients with destructive endocarditis. In cases of very severe periannular tissue destruction, the outcome does not depend on the etiological agent. Aortoventricular dehiscence, PVE, no antibiotics upon admission, and concomitant bypass surgery are strong risk factors of mortality.

**Table:** 

Authors' roles & responsibilities	
MPS	Substantial contributions to the conception or design of the work; and the acquisition, or analysis of data for the work; drafting the work; final approval of the version to be published
AW	Substantial contributions to the conception of the work; revising the work critically for important intellectual content; final approval of the version to be published
SM	Substantial contributions to the design of the work; final approval of the version to be published
AM	Substantial contributions to the design of the work; and analysis of data for the work; final approval of the version to be published
KZ	Substantial contributions to the acquisition and analysis of data for the work; final approval of the version to be published
MPBOS	Revising the work critically for important intellectual content; final approval of the version to be published
AZ	Substantial contributions to the design of the work; and acquisition of data for the work; revising the work critically for important intellectual content; final approval of the version to be published
JE	Substantial contributions to the conception of the work; and analysis of data for the work; drafting the work; final approval of the version to be published
